# Supplementary material for: Pomeranchuk instability from electronic correlations in CsTi3Bi5 kagome metal
Source: Nat Commun. 2025 Dec 23;17:325. doi: 10.1038/s41467-025-67037-4 (PMC12789546; doi:10.1038/s41467-025-67037-4)
Supplement: Supplementary file 1 — Supplementary Information [file 41467_2025_67037_MOESM1_ESM.pdf]

Supplementary Information for "Pomeranchuk instability from electronic correlations in $CsTi_3Bi_5$ kagome metal"

I. Effect of surface termination on the band structure

Comparing the bulk electronic band structure obtained from density functional theory and the recorded ARPES spectra in Fig. S1a,c, a rigid energy shift can be observed for a number of bands. To investigate the origin of this discrepancy, we perform slab calculations for a semi-infinite structure employing different surface terminations as displayed in Fig. S1b,d. We recognize, that the Cs termination induced surface band shifts are indeed compatible with the observed ARPES data confirming the validity of the utilized *ab initio* model. Since the interlayer hybridisation between the Ti kagome layer and adjacent honeycomb layers of Bi is strong, we expect the ordering tendencies at the surface of $CsTi_3Bi_5$ to be inherited from an instability in the bulk. Especially the γ band, that features the most prominent nematic ordering tendencies according to our analysis, shows a strong hybridisation between surface and bulk bands. Hence, the *ab initio* band structure for the bulk appears as a valid starting point for the subsequent many-body calculations performed in the present work.

The only feature not matched by the DFT slab calculations is given by a Dirac cone like feature at Γ around $-0.8\,\mathrm{eV}$. This has been attributed to a topological surface state with non-trivial $\mathbb{Z}_2$ invariant, that is not quantitatively accessible within surface DFT calculations even when including strong spin-orbit coupling [1]. Since this state lies well below the Fermi level and does not contribute to the nematic ordering signal, we discard it in the presented analysis.

II. Effect of spin orbit coupling and out-of-plane hybridization

A. *Electronic band structure*

The results of band structure calculations for $CsTi_3Bi_5$ both with and without enforced $SU(2)$ symmetry are juxtaposed in Fig. S2a. Close to the Fermi level, where emergent symmetry breaking is expected to happen in any weak to intermediate coupling approach, the single particle dispersion exhibits only subtle differences: while some avoided bandcrossings emerge in the presence of spin-orbit coupling (SOC), the Ti d-orbital derived bands are hardly effected, in particular the the three kagome bands formed by the in-plane orbitals of Ti (cf. Fig. 3d in the main text). Most importantly, the position of the vHs and the shape of the Fermi surface remains greatly unaltered. The only recognizable difference at low energies is the absence of the small circular (α) Fermi pocket. This can be attributed to a SOC induced self-doping effect of the Bi p_z orbital. Following Ref. [2], we consequently utilize an ab initio model with full $SU(2)$ symmetry throughout the present work and mimic the effect of SOC by introducing a local chemical potential downward shift for the p_z orbital of $\mu_{p_z} = -0.45\,\mathrm{eV}$. By this procedure the low energy band structure (grey line in Fig. S2a) is in good agreement with the SOC case. Far away from Fermi level, this simplification is no longer valid [3], but the screening effect of these bands is algebraically suppressed in the relevant susceptibilities (cf. Eq. (1) in the main text). Hence, the employed approximation is appropriate for the subsequent many-body treatment by means of the functional renormalization group.

For the employed $SU(2)$ symmetric model, we analyse the effect of k_z dispersion for a further downfolding of the model. As shown in Fig. S2b, the Fermi surface changes only slightly with k_z and most importantly does not undergo any topological transition and the Fermi surface pockets acquire a cylindrical shape in the three-dimensional Brillouin zone (cf. Fig. 3c in the main text). Inspecting the orbital resolved 3D band structure, a more differentiated picture is obtained. As expected, bands derived from in-plane Ti d-orbitals do not feature a sizable k_z dependence. Thereby, the 2D vHs at the M point preserves its logarithmically divergent density of states also in the 3D limit. Likewise, the out-of-plane d-orbital bands retain their in-plane momentum structure, but are slightly shifted to higher energies as k_z is growing. The Bi p-orbitals exhibit a more pronounced but still moderate out-of-plane dispersion.

B. *Many-body calculations*

To rule out any artifact from the 2D limit, we perform 3D FRG calculations on the full model with a momentum grid of $24 \times 24 \times 6$ $\mathbf{k}$ points, with an additional refinement of $12 \times 12 \times 5$, equipped with interactions only in the in-plane d-orbital manifold, where the electronic response is the largest (cf. Fig. 3f in the main text). We obtain a $q = 0$ Pomeranchuk instability, that provides the observed nematic deformation of the (γ) Fermi pocket. Apparently,

the absence of interactions between the out-of-plane d-orbitals heavily suppresses the nematic deformation of bands associated with these orbitals. Redoing the calculation in the 2D limit, we recover the 3D result. This evidences the 2D nature of the electronic correlations in $CsTi_3Bi_5$.

III. The choice of the interacting manifold

The considerations presented in Supplementary Information II justify a further downfolding of the model by restricting the momentum mesh for the FRG calculations to the $k_z = 0$ plane. This allows us to treat the full Ti d-orbital manifold as interacting subspace. While cRPA calculations suggest a slight increase of interactions of in-plane compared out-of-plane orbital combinations in kagome systems [4], the exact ratio is expected to be model specific. As a first order approximation, we do not considered an orbital dependency of the Kanamori parameter, since we are only interested in the qualitative changes of the ordering tendencies implied by a larger interacting manifold.

Our FRG calculations with the increased interacting subspace confirm the persistence of the Pomeranchuk instability also in the presence of additional d-orbitals, that do not feature a pronounced propensity for this order. This underlines the crucial role of the in-plane orbitals in the symmetry breaking mechanism derived from their large density of states close to the Fermi energy: they imprint their intrinsic ordering tendency on the full d-shell. Due to the strong inter-orbital interaction, the ordering on the in- and out-of-plane orbitals is strongly intertwined. This results in a comparable strength of the order parameter on all d-orbitals, when evaluating the linearised gap equation at the transition point. Inside the symmetry broken phase, where the linear approximation of the self consistency equations breaks down, we expect a sizable difference between the ordering of the in- and out-of-plane orbitals, that stems from the strong orbital polarisation of the particle-hole susceptibility (cf. Fig. 3f in the main text). Since the ARPES data is recorded deep inside the nematic phase, this expectation is in good agreement with the experimental evidence and consistent with the calculations within the in-plane orbital manifold presented in the main paper.

IV. Linear Dichroism: ARPES and DFT

In angle-resolved photoemission spectroscopy (ARPES), the measured intensity is strongly modulated by the photoemission matrix element, which introduces symmetry-based selection rules. This matrix element can be written as:

$$|M_{fi}(\mathbf{k})|^2 \propto \left|\langle \phi_f^{\mathbf{k}} | \boldsymbol{\varepsilon} \cdot \mathbf{r} | \phi_i^{\mathbf{k}} \rangle\right|^2 \tag{1}$$

where $\phi_i^{\mathbf{k}}$ and $\phi_f^{\mathbf{k}}$ denote the initial and final state wavefunctions, and $\boldsymbol{\varepsilon}$ is a unit vector pointing along the polarization direction of the incoming photon field (i.e., the vector potential $\mathbf{A}$). This expression emerges under the dipole approximation, and it implies that the ARPES intensity vanishes unless the full integrand is even with respect to the mirror plane of the system.

In typical ARPES experiments, it is reasonable to approximate the final state as a free-electron-like wavefunction with spherical symmetry, which is even under mirror reflection. Therefore, for the matrix element to be non-zero, the product $\boldsymbol{\varepsilon} \cdot \mathbf{r} | \phi_i^{\mathbf{k}} \rangle$ must also be even. Since the final state is assumed even, the character of the integrand depends primarily on the symmetry of the initial state and the dipole operator.

In our experimental setup, we define the sample's plane as the plane parallel to the crystal surface, with the in-plane directions labeled as the x and y coordinates. The z coordinate is defined as the axis perpendicular to the sample surface. The crystal possesses multiple mirror planes; however, in the context of this work, the term "mirror plane" refers specifically to the plane in which the light impinges — that is, the plane defined by the x and z coordinates.Thus, when $\boldsymbol{\varepsilon}$ lies within the mirror plane (e.g., $\boldsymbol{\varepsilon}_x$ or $\boldsymbol{\varepsilon}_z$), the operator $\boldsymbol{\varepsilon} \cdot \mathbf{r}$ is even. In this case, only initial states that are even with respect to the mirror plane contribute to the photoemission signal:

$$\boldsymbol{\varepsilon} \in \text{mirror plane} \Rightarrow \text{even operator} \Rightarrow \phi_i^{\mathbf{k}} \text{ must be even.}$$

On the other hand, when $\boldsymbol{\varepsilon}$ is oriented perpendicular to the mirror plane (e.g., $\boldsymbol{\varepsilon}_y$), the operator is odd, and only initial states with odd symmetry will contribute:

$$\boldsymbol{\varepsilon} \perp \text{mirror plane} \Rightarrow \text{odd operator} \Rightarrow \phi_i^{\mathbf{k}} \text{ must be odd.}$$

The agreement between experiment and theory is good and shown in Figure S4. Here, the calculated linear dichroism and the measured one shows a very similar agreement within the first Brillouin zone.

V. Comparison of functional renormalization group and Hartree-Fock calculations

In systems with various competing orders, a correct and unbiased assessment of the mutual competition and cooperation between the different fluctuation channels is pivotal for a truthful theoretical description. Kagome compounds provide a prime example of such systems with a rich phase diagram of unconventional magnetic, charge modulated and superconducting states [5–7]. The FRG has proven a valuable and reliable tool to investigate the ordering tendencies in the highly frustrated kagome geometry [8–11] compared to mean field or random phase approximation studies, that rely on an *a priori* selection of the favored instability channel. This roots in the intricate low energy fluctuation spectrum contributed by the van-Hove singularities in the vicinity of the Fermi level: The sublattice polarization of the van-Hove points leads to a suppression of onsite scattering channels for Fermi surface nesting events in favor of bond order (BO) fluctuations, that are expected to play a decisive role in the stabilization of exotic 2×2 charge orders in Vanadium based kagome metals [8, 12, 13]. The emergence of the 2×2 BO is hence tightly bound to the dominant nesting effects of the van-Hove points at low energy scales.

A. *Absence of bond order fluctuations and Fermi level detuning*

Also in $CsTi_3Bi_5$ the intricate sublattice structure of the low lying vHs was speculated to generate an exotic $\mathbf{q} = 0$ bond nematic order [2, 3, 14]. However, the Fermi level of $CsTi_3Bi_5$ is detuned from the vHs by $\gtrsim 10\%$ of the kagome manifold's bandwidth resulting in a destruction of sizable Fermi surface nesting effects in the FS maps of Fig. 1c in the main text. Since scattering events away from the Fermi level are algebraically suppressed by $1/\varepsilon(\mathbf{k})$, we do not expect vH scattering and the thereby driven 2×2 BO fluctuations to play a crucial role in the low energy effective theory as can be directly inferred from the missing M-peak in the bare susceptibility spectrum of Fig. 3f of the main paper. Consistently, our sophisticated many-body calculations do not show any bond character of the nematic ordering vector.

To corroborate our physical intuition for the absence of BO contributions to the ordering vector we perform FRG calculations with the same parameter set at van-Hove filling by introducing a chemical potential shift $\Delta\mu = 0.221\,\text{eV}$. The van-Hove derived hexagonal Fermi sheet Figure S5b is dominated by in-plane d-orbitals. Its strong M-point nesting directly transfers to a pronounced peak at the same transfer momentum in the susceptibility spectrum of Figure S5c. The spectrum of the particle-hole fluctuations, given by the eigenvalues of the bare susceptibility tensor $\chi^0_{\{o_i\}}$, displays peaks of comparable size at Γ and $\sim M$. The 2×2 charge fluctuations are now of comparable strength with the nematic ordering tendencies as expected for the instability spectrum of the kagome bandstructure at van-Hove filling [10]. Here we note, that Figure S5 was calculated slightly away from perfect vH filling resulting in a shift of the vH nesting peak along the KM line (compare e.g. equivalent plots for the Kagome Hubbard model in Ref. [15]). This highlights the crucial role of the chemical potential in promoting a Pomeranchuk instability in $CsTi_3Bi_5$ by disfavoring competing ordering tendencies in the system.

B. *Mean field results and absence of competing ordering vectors*

The absence of competing fluctuation channels in the FRG calculations combined with the negligible effect of the vHs on the ordering tendencies of the system questions the necessity of sophisticated many-body methods to correctly predict the nematic transition in $CsTi_3Bi_5$. To this end, we perform FRG calculations without including the cross-talk between the different diagrammatic channels. Thereby, the integration of the FRG flow equations presented in Figure 4a of the main text reduces to a infinite ladder resummation of the effective vertex in the respective interaction channels. This is equivalent to an independent random phase approximation (RPA) calculation in the superconducting, charge, and magnetic channel [16]. Since the RPA is nothing else than a saddle-point approximation of the action around the free action, the leading eigenvector of an RPA calculations resembles the minimum of the mean field free energy in the same channel (for a detailed discussion see e.g. the textbook of Nagaosa [17]). Using the same parameters as in the previous FRG calculations allows for a direct diagnosis of the effect of quantum fluctuations beyond the mean field paradigm. Our calculations show, that the nematic charge ordered state is indeed a stable minimum of the free energy already on the mean field level. This unveils the nematic order as an interaction driven phenomenon. Indeed, also in numerical studies on the kagome Hubbard model, the $\mathbf{q} = 0$ nematic charge order is emerging as the leading instability at intermediate to strong coupling [8–10]. This can be easily understood, since the charge imbalance of neighboring sites in the unit cell eases the geometrical frustration of the non-local repulsion, that is only further enhanced but not exclusively generated by higher order fluctuation processes [18].

This apparent contradiction to previous studies on $CsTi_3Bi_5$ using fluctuation exchange (FLEX) analysis in com-

bination with the density wave equation [2] can be explained quite naturally: Due to the restriction of FLEX to weak coupling and short range correlation effects, moderate spin fluctuations can emerge in an U dominated system from the bare susceptibility profile presented in Fig. 3f of the main text, that drive bond orders when projected into the charge channel. This selective renormalization of onsite spin fluctuations while neglecting a dressing of the non-local interactions, that have proven pivotal for the kagome system [12], leads to an enhancement of exotic ordering tendencies while suppressing the interaction driven MF state.

Within the FRG, a continuous interpolation between weak and intermediate coupling regime allows probe the range of validity for the interaction driven MF scenario. To do so, we record a phase diagram in the global interaction strength U and the decay behavior of the long range tale of the interaction [19]: We employ the interacting Hamiltonian presented in the Method part and use the universal scaling behavior of the screened non-local Coulomb interaction in kagome systems, that is governed by an Ohno law

$$U(\mathbf{r})/U(\mathbf{r}=0) = \frac{1}{\sqrt{r^2/\delta^2+1}} \tag{2}$$

with varying characteristic length scales δ to extract values for the nearest and next nearest interaction values V_1, V_2. Due to the computational cost of 3D simulations and baring in mind the discussion in section II we record the phase diagram in the 2D limit. Indeed we find in Figure S6, that the Pomeranchuk instability is the only abundant phase transition of the system for a large range of interactions around the physical values presented in the main paper. Reducing the interaction scale below a certain threshold results in a non-diverged flow, *i.e.* no phase transition has been encountered up to the minimal energy resolution. Dominant spin density wave fluctuations are only found within these parameter regime, where the FRG calculations are not reliable anymore due to insufficient energy resolution to continue the flow to such low scales.

Similarly, the RPA phase diagram presented in Figure S6 features the PI as only apparent symmetry breaking transition. The critical scales are generally lower compared to the corresponding FRG flows. This can be directly attributed to the missing contributions from the other channels further driving the $\mathbf{q}=0$ charge order by cross-channel feedback.

C. *Critical cutoff and estimation of the nematic transition temperature*

The phase diagram in Figure S6 the critical scale Λ_C of the Pomeranchuk instability, that serves as proxy for the expected transition temperature T_C, is highly susceptible to the choice of the initial interaction parameter and varies by several orders of magnitude in the inspected phase space. This is in line with previous investigations of the kagome Hubbard model [10] and highlights the qualitative estimate of the critical scale possible within the employed one-loop truncation of the FRG equations.

A quantitative estimate of T_C is hindered by several methodological and conceptual obstacles: Firstly, the FRG flow in the used implementation is parametrized by a sharp frequency cutoff, *i.e.* the degrees of freedom considered in the theory are limited on the imaginary Matsubara frequency axis as detailed in Ref. [20]. Thereby, no thermal broadening is employed and all calculations are nominally performed at $T=0$. Consequently, a precise conversion of Λ_C to T_C is in principle ill-defined since $T=0$.

In practice, the structure of the sharp frequency cutoff allows for interpretation of $\Lambda_C \sim T_C$. It is well known, however, that the truncation of the FRG flow equation at the four point vertex and its static approximation leads to a systematic overestimation of the critical scales, since counterterms in higher loop orders are neglected. This problem can be addressed by incorporating higher order vertices as multi-loop diagrams in the conventional FRG flow of the two particle interaction [21]. Indeed, it has been shown, that multi-loop extensions to the FRG allow for a quantitative assessment of the critical scales [22]. However, these extensions are vastly more computationally demanding and their applicability is currently limited to simple toy-models with a single orbital in the unit cell. Thereby, the presented FRG calculations within one loop approximation are not capable of providing a quantitative estimate of the expected electronic transition temperature of the Pomeranchuk instability in $CsTi_3Bi_5$.

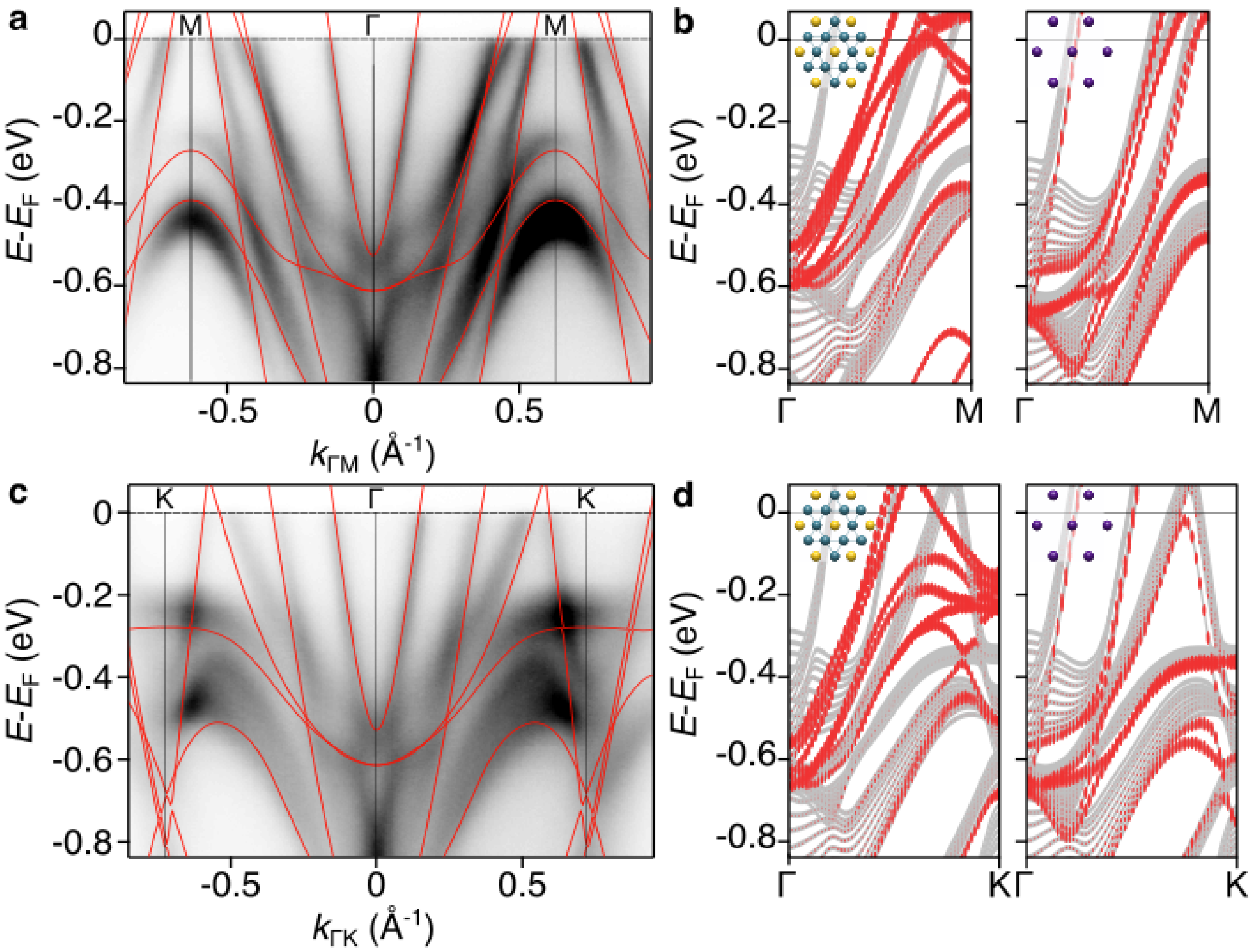

Fig. S1: **Surface-projected electronic states.** **a** Energy versus momentum dispersion along Γ-M together with the bulk, non-nematic and spinless electronic band structure (red solid lines). **b** Surface-projected calculation for the case of kagome terminated (left) and Cs-terminated (right) slabs. **c**, **d**, same for the Γ-K high-symmetry direction.

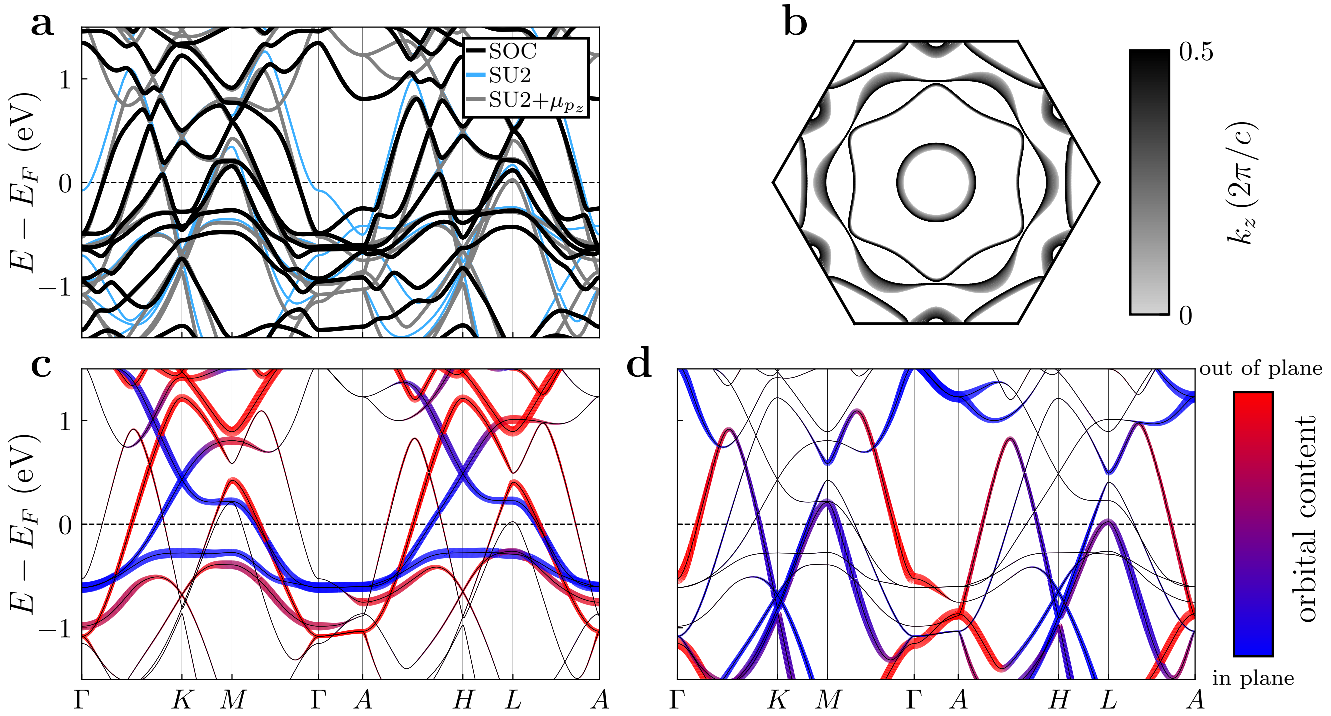

Fig. S2: **Role of SOC and $\mathbf{k}_z$ dispersion.** **a**, Comparison of the ab initio band structure with and without spin orbit coupling. The differences can be greatly reduced by introducing a chemical potential shift for the Bi-p_z orbital. **b**, 2D cuts of the Fermi level for different $\mathbf{k}_z$ values. **c**,**d**, Analogous of Fig. 3 in the main text but with the full 3D high symmetry path. While the bands with dominant Ti-d orbital contributions display little $\mathbf{k}_z$ dependence, the Bi-p orbitals acquire substantial dispersion in the out-of-plane direction.

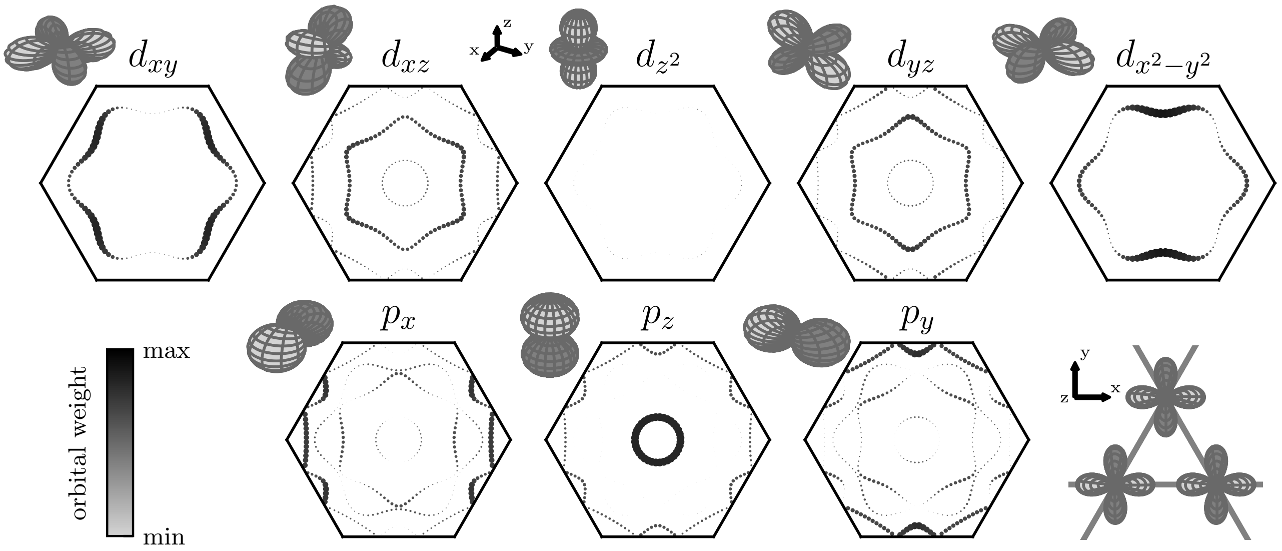

Fig. S3: **Orbital contribution on the Fermi surface.** Orbital weights $|\psi_{\mathbf{k}o}|^2$ of the electronic wavefunction ψ at the Fermi level for the Ti d and Bi p orbitals. The insets show the orbital basis used to construct the TB model. The employed atomic orbital basis features a global quantization axis in real space as exemplified for the Ti $d_{x^2-y^2}$ orbital in the lower right panel.

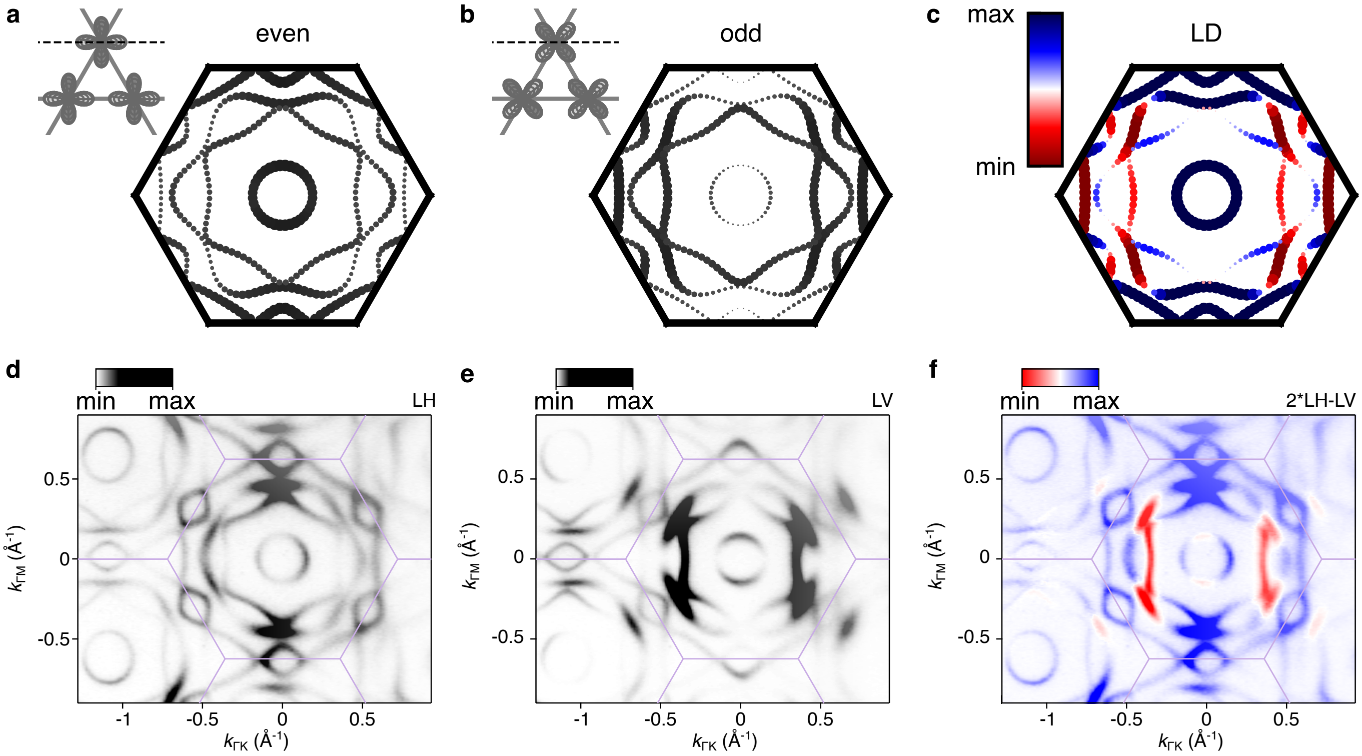

Fig. S4: **Orbital parity of the Fermi surface and linear dichroism.** **a**, **b** Collected Fermi surface weight of orbitals with even and odd parity w.r.t. the mirror indicated by a dashed line in the inset. These are Ti $d_{x^2-y^2,z^2,xz}$ Bi $p_{x,z}$ and Ti $d_{xy,yz}$, Bi p_y, respectively. **c** The amplitude difference between both contributions (even - odd) gives an estimate of the dominant parity along the Fermi surface segment. The results are in good agreement with the Fermi contours measured in the ΓKM plane (hν=65 eV) with **d** linear horizontal and **e** linear vertical light polarizations. The amplitude difference between both contributions (right panel) is consistent with the experimental linear dichroism (LD) pattern displayed in **f**.

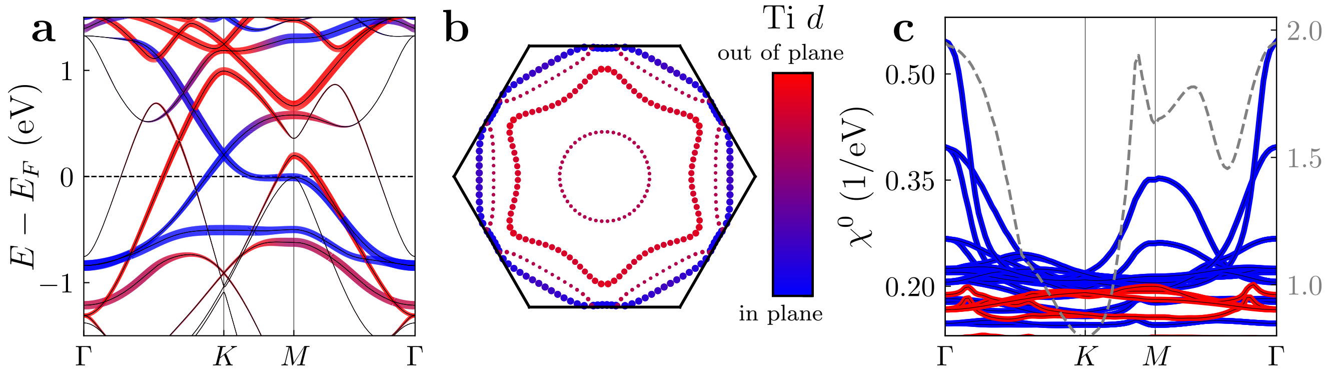

Fig. S5: **Electronic fluctuation spectrum at van-Hove filling.** The orbital resolved bandstructure (**a**) and Fermi surface (**b**) close to van-Hove filling are obtained by a chemical potential shift of $\Delta\mu = 0.221\,\mathrm{eV}$. The orbital resolved bare susceptibility (**c**) shows a peak at $\mathbf{q} = M$ in accordance with the dominant FS nesting vector. The leading eigenvalue of $\chi^0(\mathbf{q})$ (dashed grey) shows a comparable strength of Γ and M-point fluctuations.

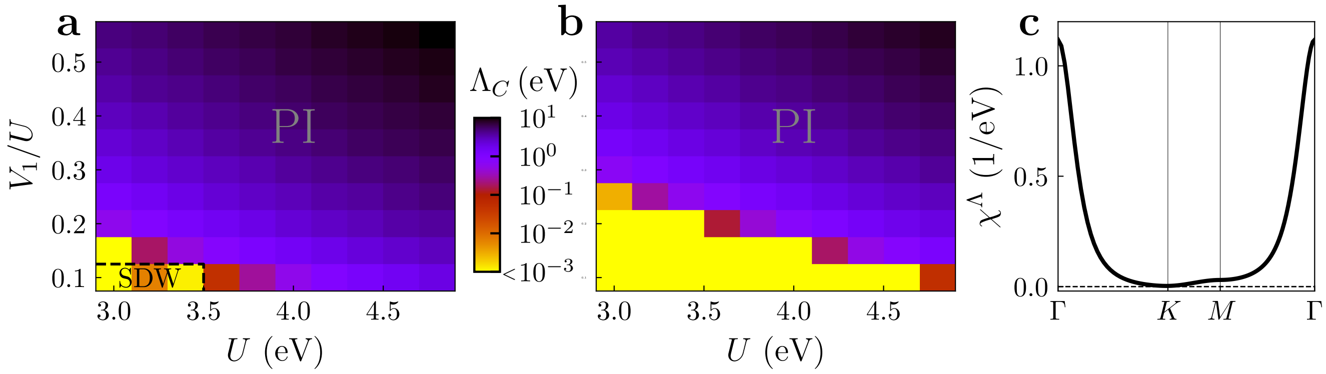

Fig. S6: **FRG and RPA phase diagrams.** (**a**) We calculate the FRG flow for the parameter set given in the methods section. We employ the interaction tensor given by Eq. (2) in the main paper with fixed $J = 0.2U$ and vary the onsite repulsion U and the long range decay of the interaction governed by Equation 2. From this equation, V_2 is directly given by the corresponding value of V_1, that is indicated on y-axis. The color indicates the critical scale, at which a divergent scattering vertex was encountered within the FRG. Besides the abundant Pomeranchuk instability (PI), a spin-density wave (SDW) was only encountered at very low scales around the energy resolution of the calculation ($\sim 10^{-3}$ eV), where the calculations are not reliable anymore. (**b**) We perform the same parameter scan when turning off cross-channel projections. This is equivalent to an RPA (and hence MF) analysis in the three diagrammatic channels. Again the PI is the only prevalent phase transition in the system. The scales are generically lowered due to the missing cross-talk between the channels. (**c**) Leading eigenvalue of the RPA charge susceptibility for $U = 4\,\text{eV}$ and $V_1 = 1.5\,\text{eV}$ and $V_2 = 0.5\,\text{eV}$ (same parameters as for the FRG flow in Fig. 4b of the main paper) close to the divergence at $\Lambda = 2.5\,\text{eV}$. The nematic fluctuations at $\mathbf{Q} = \Gamma$ are close to criticality before quantum fluctuations related to the Fermi surface nesting visible in Fig. 3f can imprint competing fluctuation channels to the susceptibility.

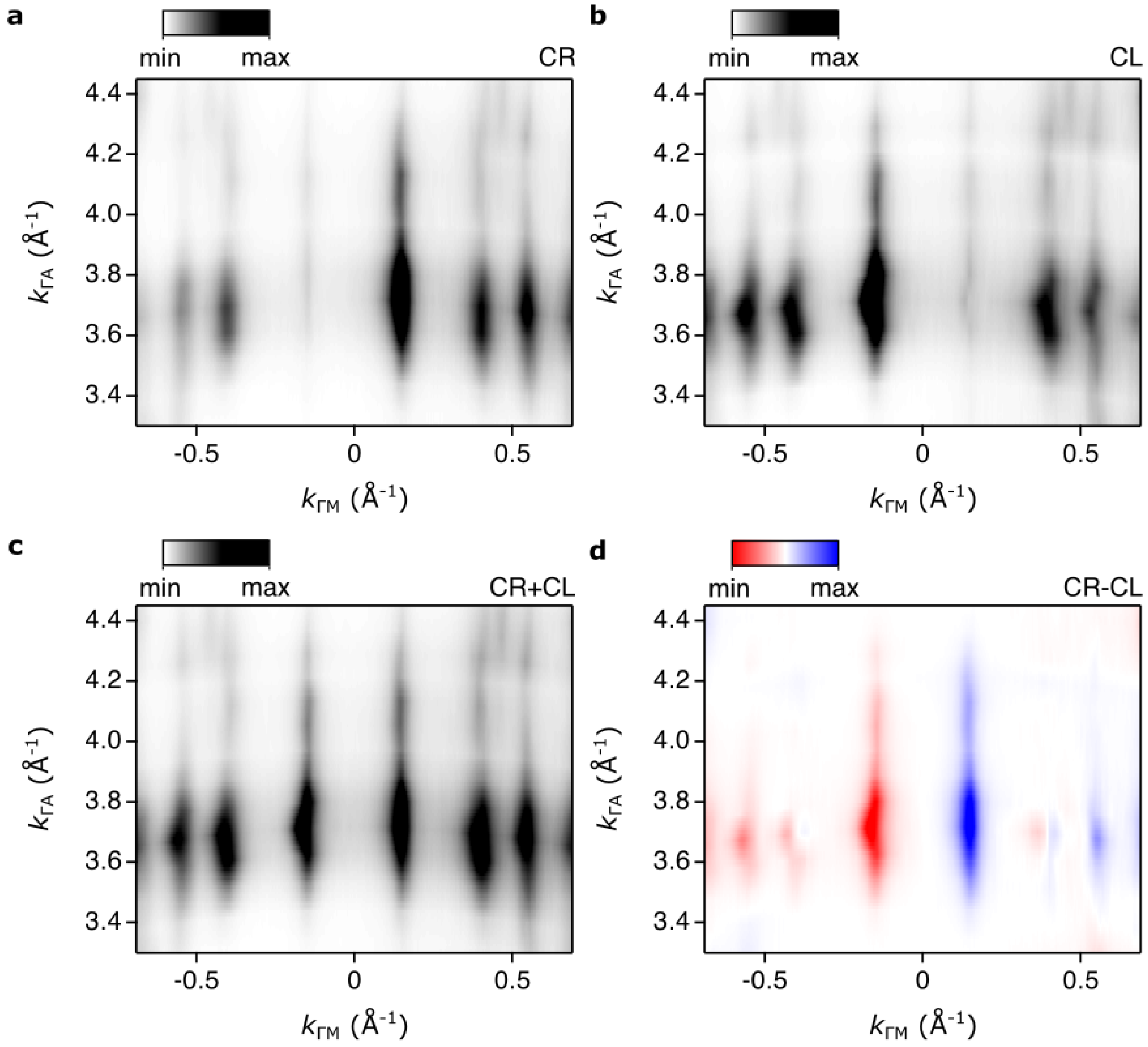

Fig. S7: k_z **versus** $k_{||}$ **at the Fermi level.** . The k_z-maps are obtained by scanning hν from 40 eV to 70 eV with both **a** Circular Right (CR) and **b** Circular Left (CL) helicities. **c** unpolarised light obtained by summing up the k_z maps collected with right- and left-handed circularly polarised light and **d** the respective circular dichroism. Sample is oriented with the Γ-M direction along the analyser's slit.

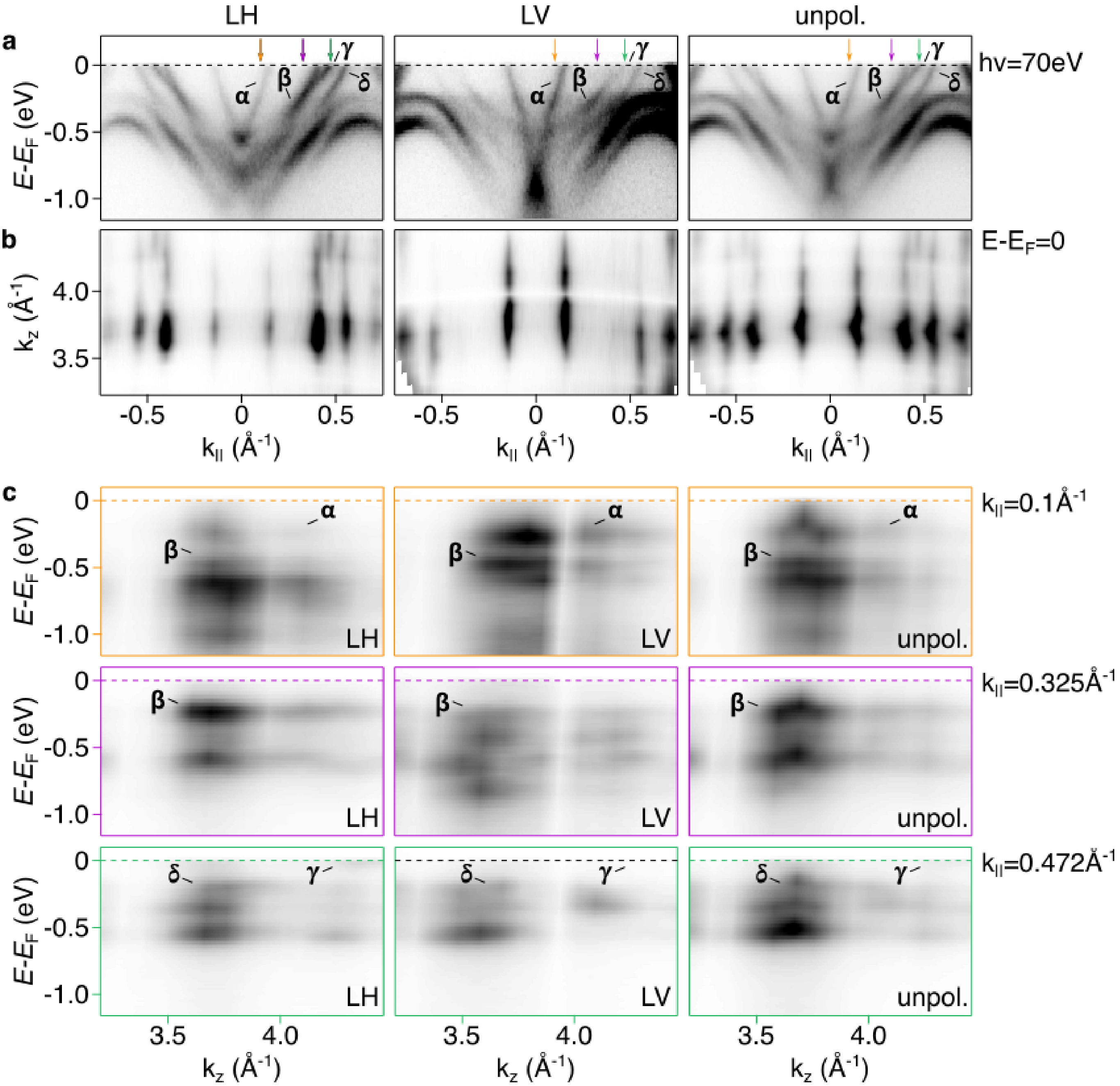

Fig. S8: **Band structure two-dimensional character.** **a**, Band dispersion probed with 70 eV photon energy. **b**, k_z-maps at the Fermi level obtained by scanning $h\nu$ from 32.5 eV to 70 eV. **c**, The absence of striking band dispersion along k_z has been sampled at different $k_{||}$ points (marked by coloured arrows in panel **a**) is consistent with the calculated two-dimensional character of the Fermi contour. To rule out geometrical and matrix element effects, we used both linear horizontal, linear vertical and unpolarised (CR+CL) light polarisations. Sample is oriented with the Γ-M direction along the analyser's slit. The relevant bands building the Fermi contours are labelled following the same notation of the main text.

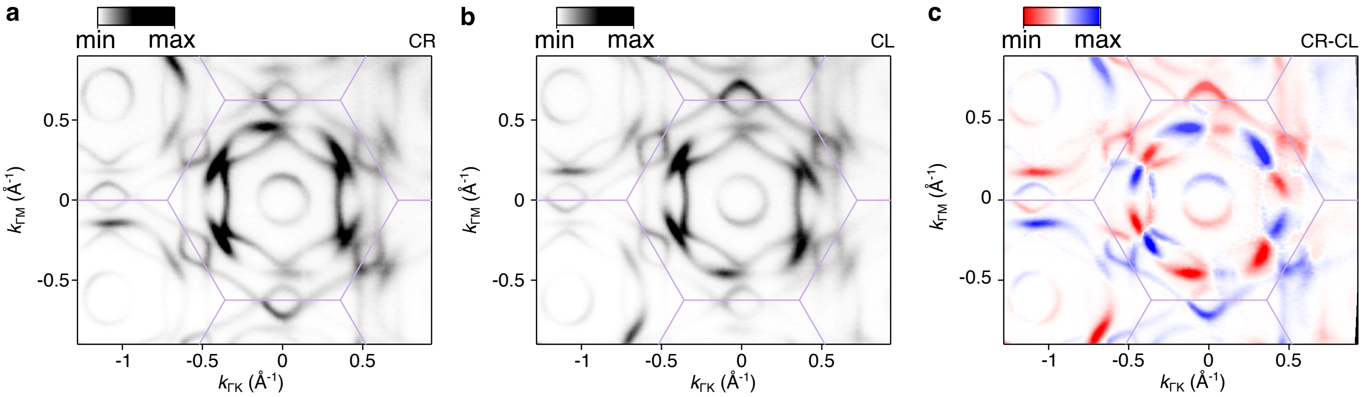

Fig. S9: **Polarisation dependence of the Fermi contours.** Fermi surface contours measured in the ΓKM plane (hν=65 eV) with different light polarisations: **a** circular right, **b** circular left and **c** the resulting circular dichroism.

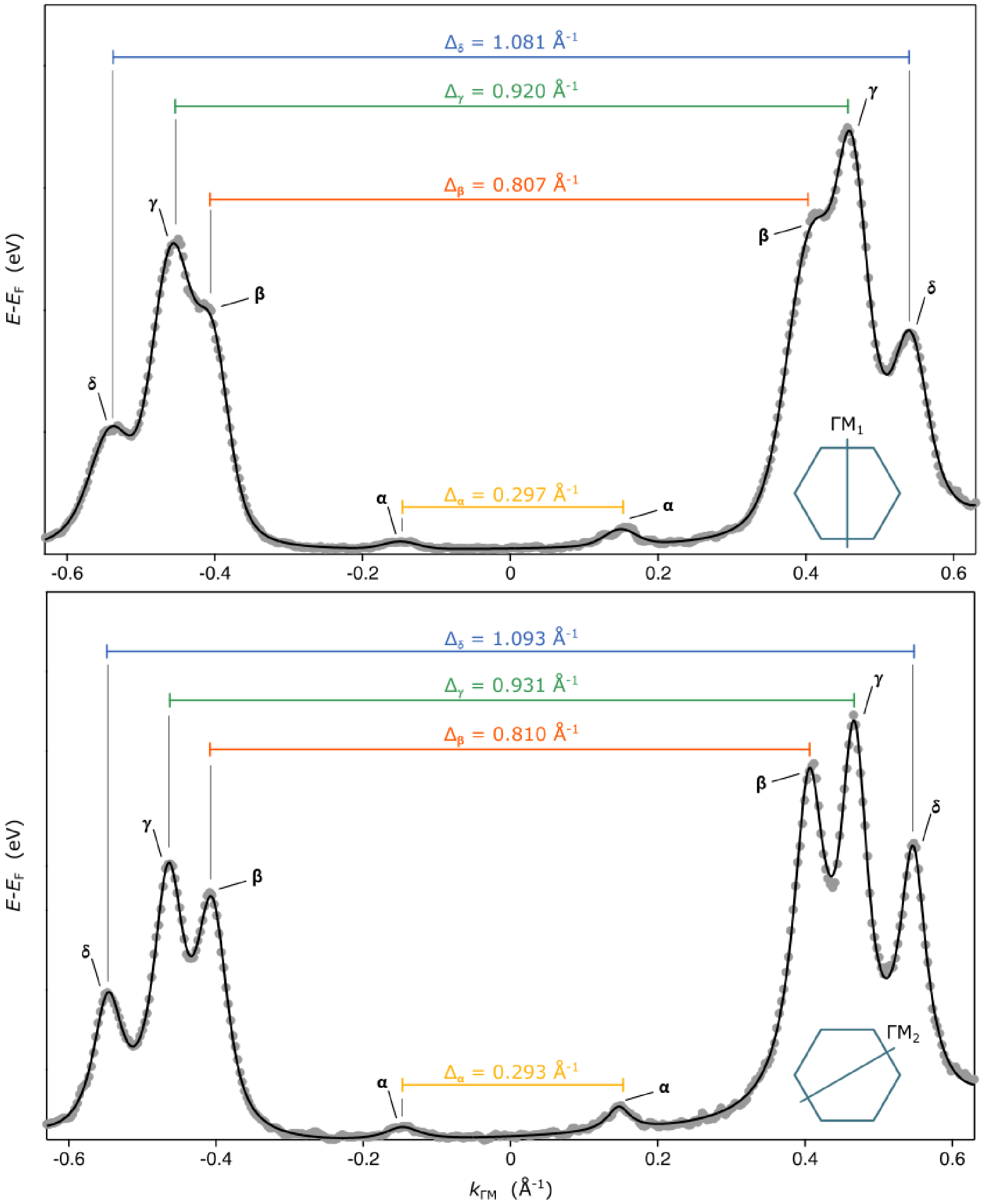

Fig. S10: **Lorentzian fit at the Fermi level.** Momentum distribution curves (MDCs) extracted at the Fermi level along two inequivalent Γ-M directions (LH) to show the changes in the fermi momenta of (γ) and (δ) bands. Grey dots are the data points, and the black solid line is the respective fit. The fit, to capture all the bands, was done on data collected by using LH polarization.

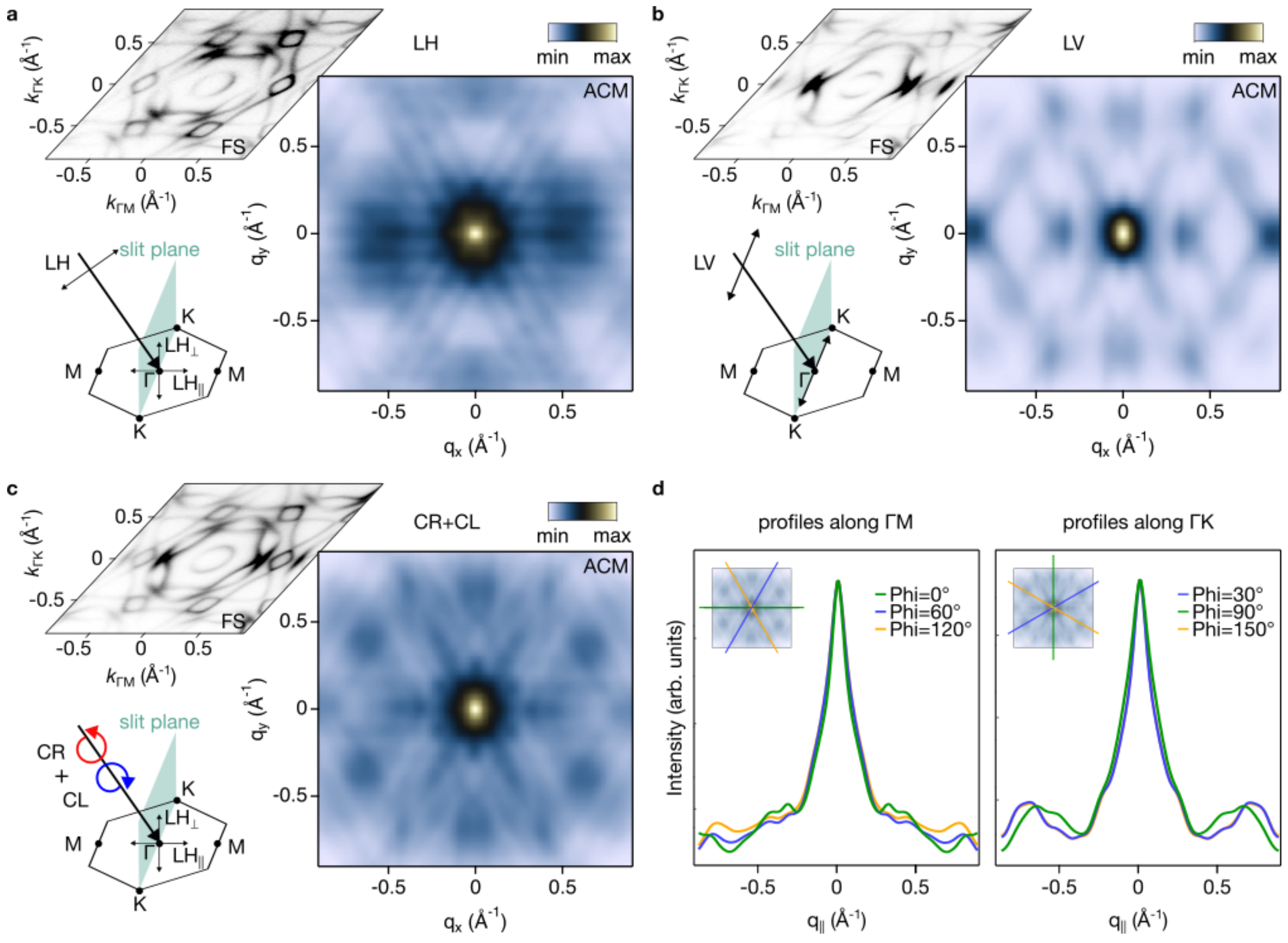

Fig. S11: **Electronic nematicity of the Fermi contour.** Analogous of Figure 2 of the main text but with Γ-K high-symmetry direction aligned along the analyser's slit. **a**, Autocorrelation map (ACM) extracted at the Brillouin zone centre with $h\nu = 65$ eV for linearly horizontal polarised light, **b** linear vertical polarisation and **c** unpolarised light obtained by summing up spectra collected with right- and left-handed circularly polarised light. The cartoons illustrate the experimental geometry and vector projections. **d**, Azimuthal profiles (extracted from the ACM in **c**) emphasize the reduced C_2 symmetry.

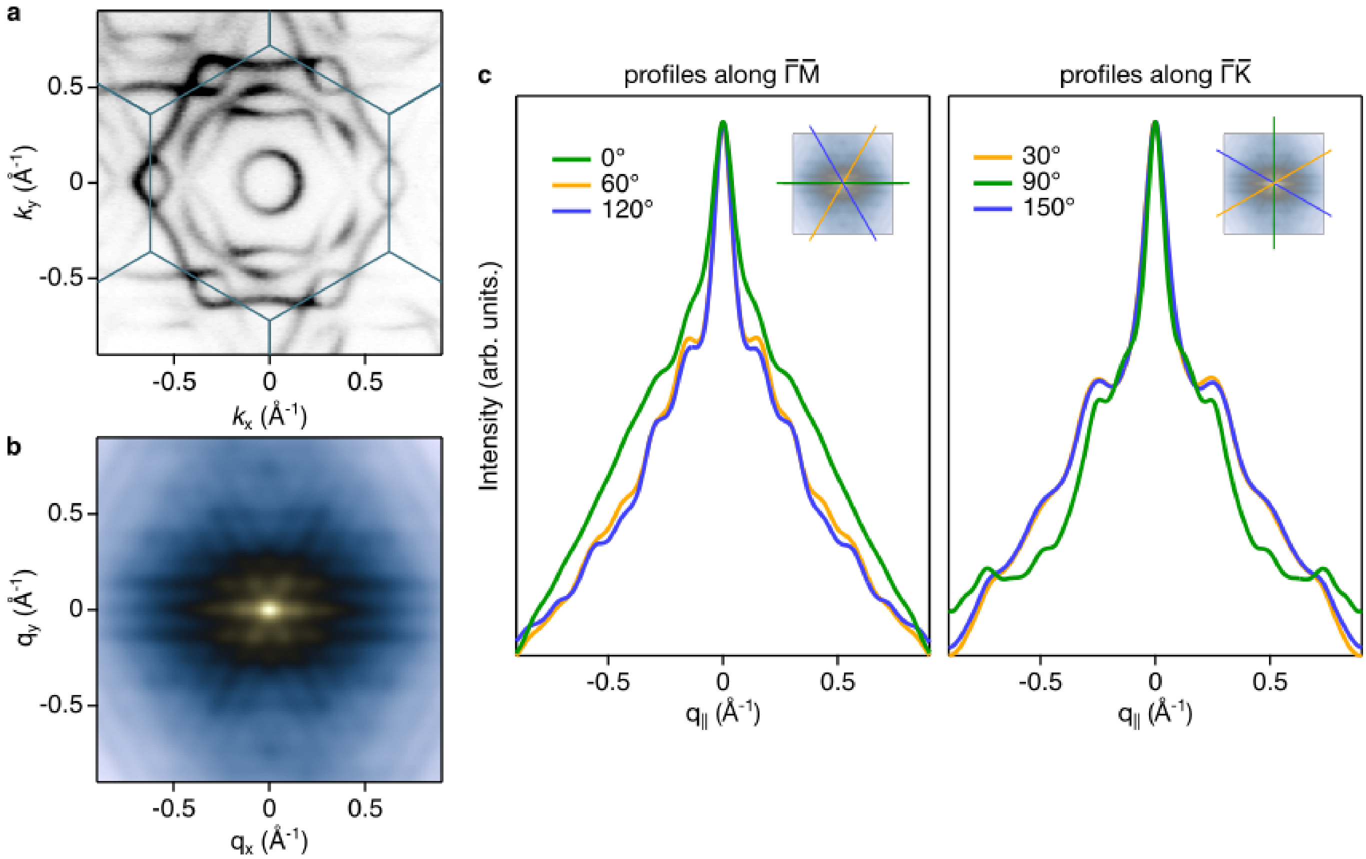

Fig. S12: **Electronic nematicity at finite k_z.** **a**, Fermi surface contour obtained with 75 eV linear horizontal polarisation light, *i.e.* away from the Brillouin zone center $k_z = 0$. The sample is oriented with the Γ-K direction along the analyser's slit. **b**, The associated autocorrelation map. **c**, Azimuthal profiles extracted from the ACM in **c**, supporting the reduced nematic C_2 symmetry.

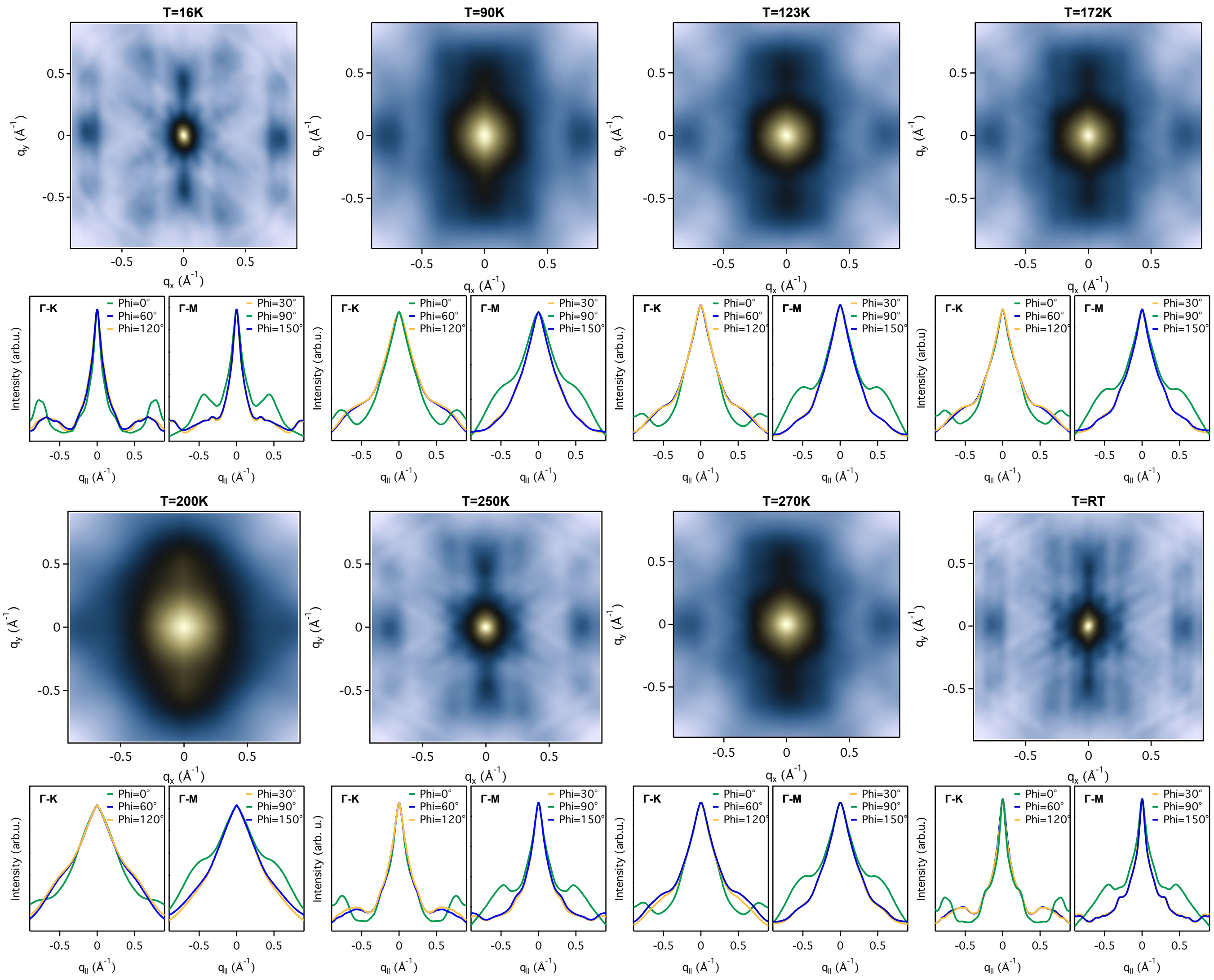

Fig. S13: **Temperature dependent ARPES.** Autocorrelation maps obtained from the Fermi surface contours of unpolarised light (CR+CL) probed at the Brillouin zone centre with hν = 65 eV and for several temperatures ranging from 16 K up to room temperature. The azimuthal profiles extracted for the Γ-K and Γ-M high-symmetry directions clearly show the persistence of the reduced C_2 symmetry of the nematic phase up to room temperature. Importantly, variations in broadness and in the profile shapes for different temperature points (e.g. for the 200 K set) are ascribable to the sample aging and causality of the cleave rather than the onset of a transition: if a cleave is not ideal and perfectly flat, electron-impurity scattering is induced. As a consequence the self energy acquires a sizable imaginary part causing a blurring of the signal independent of temperature effects in exfoliable crystals.

[1] J. Yang, X. Yi, Z. Zhao, Y. Xie, T. Miao, H. Luo, H. Chen, B. Liang, W. Zhu, Y. Ye, et al., Nature Communications **14**, 4089 (2023), URL https://doi.org/10.1038/s41467-023-39620-0.
[2] J. Huang, Y. Yamakawa, R. Tazai, T. Morimoto, and H. Kontani, arXiv:2305.18093 (2023).
[3] Y. Hu, C. Le, Y. Zhang, Z. Zhao, J. Liu, J. Ma, N. C. Plumb, M. Radovic, H. Chen, A. P. Schnyder, et al., Nature Physics **19**, 1827 (2023), ISSN 1745-2481, URL https://doi.org/10.1038/s41567-023-02215-z.
[4] D. Di Sante, B. Kim, W. Hanke, T. Wehling, C. Franchini, R. Thomale, and G. Sangiovanni, Phys. Rev. Res. **5**, L012008 (2023), URL https://link.aps.org/doi/10.1103/PhysRevResearch.5.L012008.
[5] H. Zhao, H. Li, B. R. Ortiz, S. M. L. Teicher, T. Park, M. Ye, Z. Wang, L. Balents, S. D. Wilson, and I. Zeljkovic, Nature **599**, 216 (2021), URL https://doi.org/10.1038/s41586-021-03946-w.
[6] T. Neupert, M. M. Denner, J.-X. Yin, R. Thomale, and M. Z. Hasan, Nature Physics **18**, 137 (2022), ISSN 1745-2481, URL https://doi.org/10.1038/s41567-021-01404-y.
[7] C. Guo, G. Wagner, C. Putzke, D. Chen, K. Wang, L. Zhang, M. Gutierrez-Amigo, I. Errea, M. G. Vergniory, C. Felser, et al., Nature Physics **20**, 579 (2024), ISSN 1745-2481, URL https://doi.org/10.1038/s41567-023-02374-z.
[8] M. L. Kiesel, C. Platt, and R. Thomale, Phys. Rev. Lett. **110**, 126405 (2013), URL https://link.aps.org/doi/10.1103/PhysRevLett.110.126405.
[9] W.-S. Wang, Z.-Z. Li, Y.-Y. Xiang, and Q.-H. Wang, Phys. Rev. B **87**, 115135 (2013), URL https://link.aps.org/doi/10.1103/PhysRevB.87.115135.
[10] J. B. Profe, L. Klebl, F. Grandi, H. Hohmann, M. Dürrnagel, T. Schwemmer, R. Thomale, and D. M. Kennes, Phys. Rev. Res. **6**, 043078 (2024), URL https://link.aps.org/doi/10.1103/PhysRevResearch.6.043078.
[11] T. Schwemmer, H. Hohmann, M. Dürrnagel, J. Potten, J. Beyer, S. Rachel, Y.-M. Wu, S. Raghu, T. Müller, W. Hanke, et al., Phys. Rev. B **110**, 024501 (2024), URL https://link.aps.org/doi/10.1103/PhysRevB.110.024501.
[12] M. L. Kiesel and R. Thomale, Phys. Rev. B **86**, 121105 (2012), URL https://link.aps.org/doi/10.1103/PhysRevB.86.121105.
[13] R. Tazai, Y. Yamakawa, and H. Kontani, Nature Communications **14**, 7845 (2023), URL https://doi.org/10.1038/s41467-023-42952-6.
[14] H. Li, S. Cheng, B. R. Ortiz, H. Tan, D. Werhahn, K. Zeng, D. Johrendt, B. Yan, Z. Wang, S. D. Wilson, et al., Nature Physics **19**, 1591 (2023), URL https://doi.org/10.1038/s41567-023-02176-3.
[15] A. T. Rømer, S. Bhattacharyya, R. Valentí, M. H. Christensen, and B. M. Andersen, Phys. Rev. B **106**, 174514 (2022), URL https://link.aps.org/doi/10.1103/PhysRevB.106.174514.
[16] A. Fischer, L. Klebl, J. B. Profe, A. Rothstein, L. Waldecker, B. Beschoten, T. O. Wehling, and D. M. Kennes, Phys. Rev. Res. **6**, L012003 (2024), URL https://link.aps.org/doi/10.1103/PhysRevResearch.6.L012003.
[17] N. Nagaosa, *Quantum Field Theory in Condensed Matter Physics*, Theoretical and Mathematical Physics (Springer, 1999), ISBN 978-3-540-65537-4, 978-3-642-08485-0, 978-3-662-03774-4.
[18] J. Zhan, H. Hohmann, M. Dürrnagel, R. Fu, S. Zhou, Z. Wang, R. Thomale, X. Wu, and J. Hu, arXiv:2506.01648 (2025).
[19] D. Di Sante, C. Bigi, P. Eck, S. Enzner, A. Consiglio, G. Pokharel, P. Carrara, P. Orgiani, V. Polewczyk, J. Fujii, et al., Nature Physics **19**, 1135 (2023), URL https://doi.org/10.1038/s41567-023-02053-z.
[20] J. B. Profe, D. M. Kennes, and L. Klebl, SciPost Phys. Codebases p. 26 (2024), URL https://scipost.org/10.21468/SciPostPhysCodeb.26.
[21] F. B. Kugler and J. von Delft, Phys. Rev. Lett. **120**, 057403 (2018), URL https://link.aps.org/doi/10.1103/PhysRevLett.120.057403.
[22] C. Hille, F. B. Kugler, C. J. Eckhardt, Y.-Y. He, A. Kauch, C. Honerkamp, A. Toschi, and S. Andergassen, Phys. Rev. Res. **2**, 033372 (2020), URL https://link.aps.org/doi/10.1103/PhysRevResearch.2.033372.
